# Construction and expression of D-dimer and GPIIb/IIIa single-chain bispecific antibody

**DOI:** 10.3892/etm.2013.1132

**Published:** 2013-05-30

**Authors:** ZHAOKUI DAN, ZUI TAN, HONGLI XIA, GAN WU

**Affiliations:** 1 Department of Vascular Surgery, Zhongnan Hospital of Wuhan University, Wuhan, Hubei 430071;; 2 Department of Hepatobiliary Gastrointestinal Surgery, Xianning Central Hospital, Xianning, Hubei 437100;; 3 Centre of Inspection, Detection and Testing on GMO Environmental Biosafety, The Ministry of Agriculture, Wuhan, Hubei 430062, P.R. China

**Keywords:** D-dimer, glycoprotein IIb-IIIa, single-chain bispecific antibody

## Abstract

The aim of this study was to construct a plasmid expressing glycoprotein IIb-IIIa (GPIIb/IIIa) and D-dimer single-chain bispecific antibody for the targeted therapy of thrombosis. The phosphorylated gene encoding the anti-GPIIb/IIIa single-chain variable fragment (scFv) and the gene encoding the anti-D-dimer scFv were amplified by PCR and linked in tandem by blunt-end ligation. The recombinant plasmid was transfected into the competent cell line HB2151 and identified by PCR and DNA sequencing. Then, the soluble recombinant antibody in bacterial lysates was purified by an NTA column and molecular sieve chromatography in turn. Finally, the binding specificity of the purified antibody was tested by enzyme-linked immunosorbent assay (ELISA). Results demonstrated that the construction of the expression plasmid was successful and the purified recombinant protein, which had a molecular weight of ∼56 kDa, was specific to GPIIb/IIIa and D-dimer. In conclusion, a plasmid expressing a bispecific antibody was constructed by a new method of blunt-end ligation. The soluble recombinant protein is a promising platform for target-oriented thrombolytic therapy.

## Introduction

Cross-linked fibrin and activated platelets constitute the main components of a thrombus (1). Moreover, the activation of platelet glycoprotein IIb/IIIa (GPIIb/IIIa), which is abundantly expressed on the platelet surface (2), is the final common pathway of platelet aggregation (3). Therefore, fibrin, the fibrin degradation product (D-dimer) and GPIIb/IIIa may be used as targets in thrombolysis. Since single-chain urokinase plasminogen activator (scu-PA) was covalently linked to the Fab’ region of a monoclonal antibody specific for fibrin (antibody 59D8) by Bode *et al* (4), targeted thrombolytics have become a popular research topic. Targeted thrombolytics are synthesized by connecting thrombus-specific antibodies to thrombolytic drugs via chemical or biological methods, thus producing a new type of drug with high avidity and specificity for the thrombus. This may reduce its reaction with non-target tissues.

A single-chain variable fragment (scFv), which retains the specificity of the original immunoglobulin, is a fusion protein of the variable regions of the heavy (VH) and light (VL) chains of immunoglobulins connected to a linker peptide (5). In previous studies, our research group has successfully isolated specific human monoclonal anti-D-dimer scFv antibodies (6) and monoclonal anti-GPIIb/IIIa scFv antibodies from scFv phage libraries (7); the two scFv fragments were produced in *Escherichia coli* in soluble forms with good retention of antigen-binding activities. Previously, researchers devised methods for linking two scFvs to produce a single peptide chain with two VH and two VL regions, yielding bispecific scFvs (bs-scFvs) with a specificity for two different antigens (8,9). Therefore, in this study, we used the plasmids of anti-D-dimer scFv and anti-GPIIb/IIIa scFv to construct a prokaryotic plasmid expressing GPIIb-IIIa and D-dimer bs-scFvs. The single-chain diabody binds two specific antigens simultaneously and may remarkably improve specificity and functional avidity to a thrombus; therefore, it lays a sound foundation for further research on target-oriented thrombolytics.

## Materials and methods

### Materials

**Figure 1.**
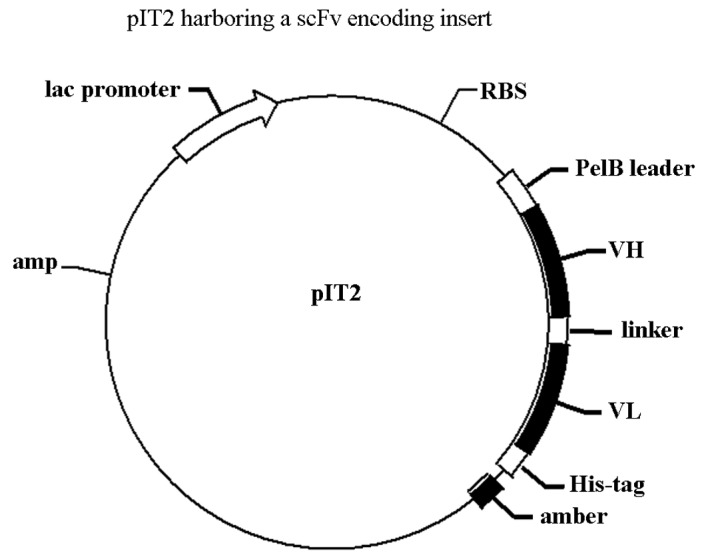
Map of vector pIT2 harboring a single-chain variable fragment (scFv)-encoding insert. RBS, ribosome binding site; PelB, signal sequence; VH-linker-VL, scFv; His-tag, immunopurification tag. A TAG amber stop codon was present at the junction of the scFv gene and gIII. The presence of an amber stop codon allows the production of scFv molecules as soluble antibody molecules instead of scFv-pIII fusion proteins. The size of the empty vector pIT2 was 4.2 kb, while the size of the scFv insert was ∼750 bp.

The human anti-D-dimer scFv component, designated A1, and the human anti-GPIIb-IIIa scFv component, designated G9, which were previously isolated from a human scFv phage display library, were employed as fusion partners for the creation of a bs-scFv. The two scFvs were assembled in a VH-to-VL orientation, where the V-domains were attached by a 15 amino acid residue linker of composition (Gly_4_Ser)_3_, which did not interfere with antigen binding (Fig. 1). The gene sequences of A1-scFv and G9-scFv have been determined previously (6,7,). The primers were synthesized by Tsingke Biotechnology Co., Ltd. (Beijing, China). The primers are shown in Table I; the primers named linker^+^vlb^+^ and vlb^−^ were phosphorylated at the 5′ end. KOD Plus High Fidelity DNA polymerase was purchased from Toyobo Co., Ltd. (Osaka, Japan). T4 DNA ligase was purchased from New England Biolabs (Ipswich, MA, USA). The NTA column was purchased from Merck KGaA (Darmstadt, Germany). All other reagents were domestically produced biochemical analytical reagents.

### Vector construction

**Figure 2.**
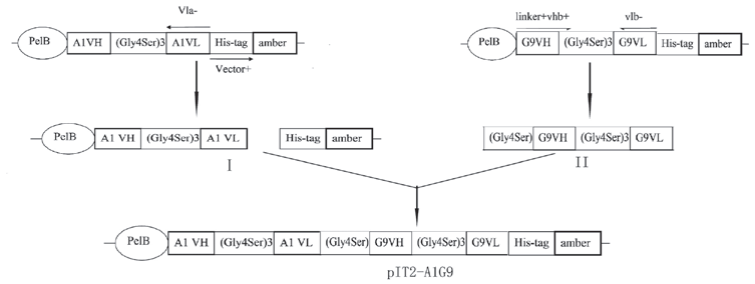
Design and various cloning steps leading to the final bispecific construct.

Taking the anti-D-dimer circular plasmid as a template, PCR was performed using primers vla^−^ and Vector^+^ to obtain a linear plasmid, named construct I (Fig. 2) with two blunt ends. To introduce the Gly_4_Ser linker at the 5′ end, PCR fragments with two blunt ends were generated (construct II) via two primers: linker^+^vlb^+^ and vlb^−^. All the above PCR fragments were amplified with KOD Plus High Fidelity DNA polymerase. Following identification by agarose gel electrophoresis, constructs I and II (Fig. 2) were retrieved and purified using the QIA Quick Extraction kits. Then, constructs I and II were linked together using T4 DNA ligase to generate the recombinant circular plasmid of anti-D-dimer/anti-GPIIb-IIIa diabody, designated pIT2-A1G9. The generation procedure of pIT2-A1G9 is described in Fig. 2. Following the transfection of pIT2-A1G9 into the competent cell line HB2151, the recombinant clone was selected from the ampicillin agar plate and characterized by PCR. Next, the clones with the correct insertion sequences were determined by sequencing.

### Bacterial expression

A single recombinant colony was selected and inoculated overnight in 5 ml Luria-Bertani (LB) culture solution containing 100 mg/ml ampicillin. Then, the colony was transferred to 250 ml LB medium containing 100 mg/ml ampicillin and inoculated to an OD600 of 1.0. Isopropylthio-β-galactoside (IPTG) was added to a final concentration of 0.4 mM and growth was continued at 37°C for 3.5 h. Cells were harvested by centrifugation at 1,800 × g for 30 min and frozen at −20°C. Following resuspension in 20 mM Tris-HCl and 0.2 M NaCl (pH 8.0), cells were broken by a high pressure homogenizer and centrifugation at 12,000 × g for 30 min. Then, the supernatants containing the soluble product were analyzed by sodium dodecyl sulfate-polyacrylamide gel electrophoresis (SDS-PAGE).

### Purification of the recombinant protein

The supernatants containing the recombinant antibody were purified using the NTA column. The bound proteins were eluted with a gradient of imidazole from 30 to 500 mM, then the fractions were analyzed by SDS-PAGE. Elution occurred at a concentration of 30 mM imidazole as a distinct peak. Then, 30 mM imidazole eluate containing the recombinant diabody was further purified with 10 mM phosphate-buffered saline (PBS) by gel filtration chromatography (HiPrep^™^ 16/60 Sephacryl S-200 high resolution column). All eluted fractions were collected and measured at 280 nm for protein.

### Binding specificity of the recombinant antibody

After the ELISA plates were coated with 25 *μ*g/ml bovine serum albumin (BSA; negative control), 25 *μ*g/ml GPIIb/IIIa or 25 *μ*g/ml D-dimer, respectively, 50 *μ*l purified recombinant antibody (20 *μ*g/ml in PBS) was added and detected with a 1:5,000 dilution of peroxidase-conjugated anti-6X histidine. The absorbance values were measured following the addition of the substrate.

## Results

### Successful construction of bispecific single-chain molecules

**Figure 3.**
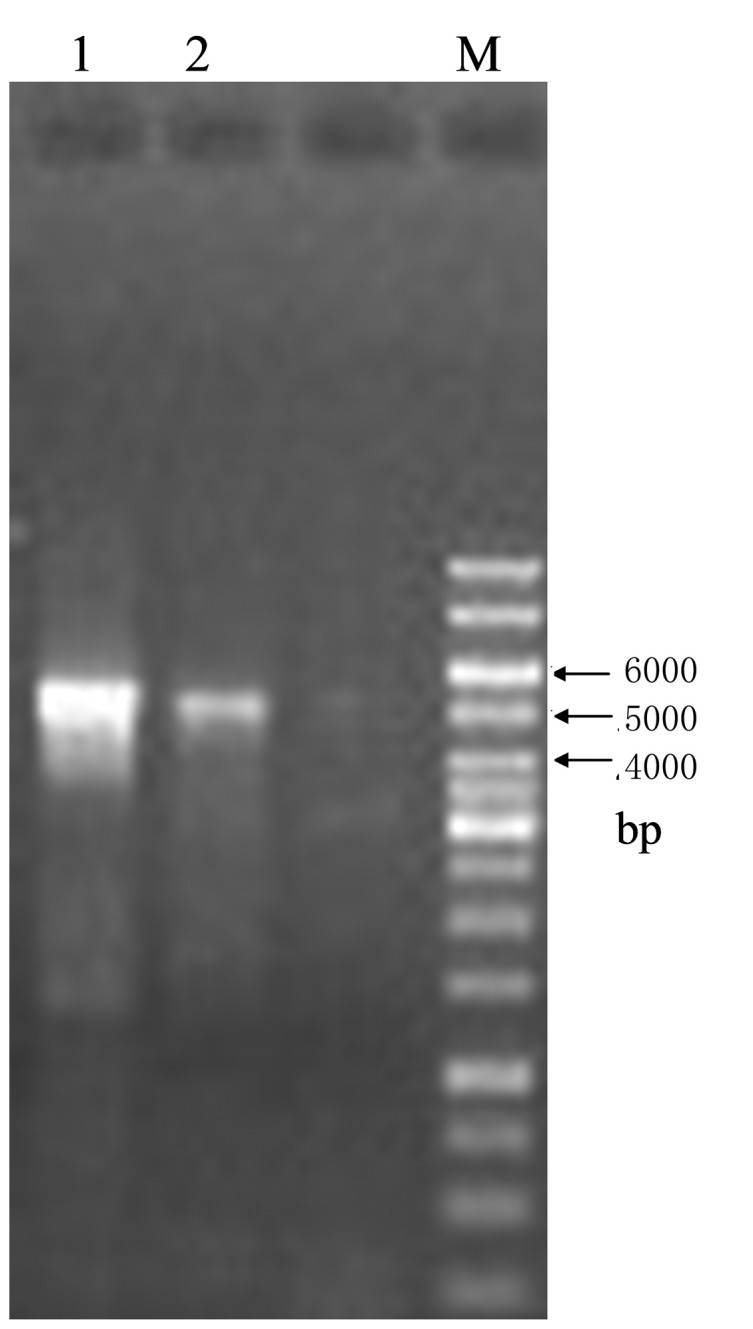
Agarose gel electrophoresis of purified construct I. Lane 1, linear construct I (∼4,950bp); lane 2, duplicate; lane M, O’GeneRuler™ 1 kb DNA Ladder.

**Figure 4.**
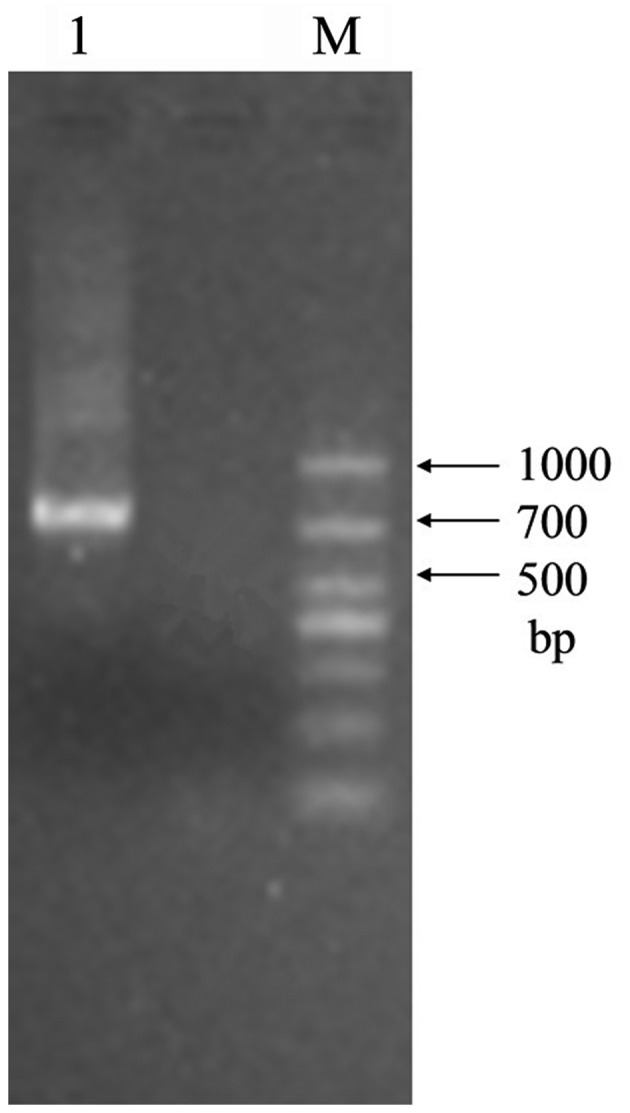
Agarose gel electrophoresis of purified construct II. Lane 1, construct II (∼750 bp); lane M, DL2000 DNA Marker.

For the generation of bispecific D-dimer GPIIb-IIIa single-chain constructs, the anti-D-dimer scFv fragments (named A1-scFv) and anti-GPIIb-IIIa scFv fragments (named G9-scFv) were fused into a tandem using a five amino acid residue glycine-serine linker (Fig. 1), which was considered to prevent the formation of scFv molecules from the adjacent A1VL and G9VH domains. The size of the empty vector pIT2 was 4.2 kb, while the size of the scFv insert was ∼750 bp. The expected sizes of construct I and construct II were ∼4,950 bp and ∼750 bp, respectively. Agarose gel electrophoresis confirmed that the actual sizes of purified construct I and construct II were in line with the expectations (Figs. 3 and 4). The recombinant clone in the correct A1VH-A1VL-G9VH-G9VL orientation was selected from the ampicillin agar plate and underwent PCR and sequencing (figure not shown).

### Expression and purification of the recombinant protein

**Figure 5.**
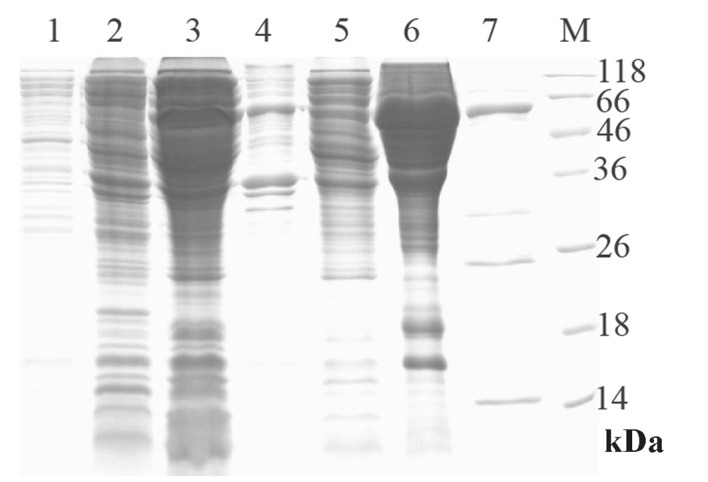
Expression and purification of the diabody (10% SDS-PAGE). Lane 1, total protein of bacterial pIT2-A1G9 prior to induction; lane 2, total protein of bacterial pIT2-A1G9 induced with IPTG; lane 3, periplasmic lysates of bacterial pIT2-A1G9 induced with IPTG; lane 4, cell lysate precipitate of bacterial pIT2-A1G9 induced with IPTG; lane 5, the Ni-NTA column flow-through; lane 6, the eluate washed at a concentration of 30 mM imidazole; lane 7, the eluate washed at a concentration of 500 mM imidazole; lane M, protein marker. SDS-PAGE, sodium dodecyl sulfate-polyacrylamide gel electrophoresis; IPTG, isopropylthio-β-galactoside.

**Figure 6.**
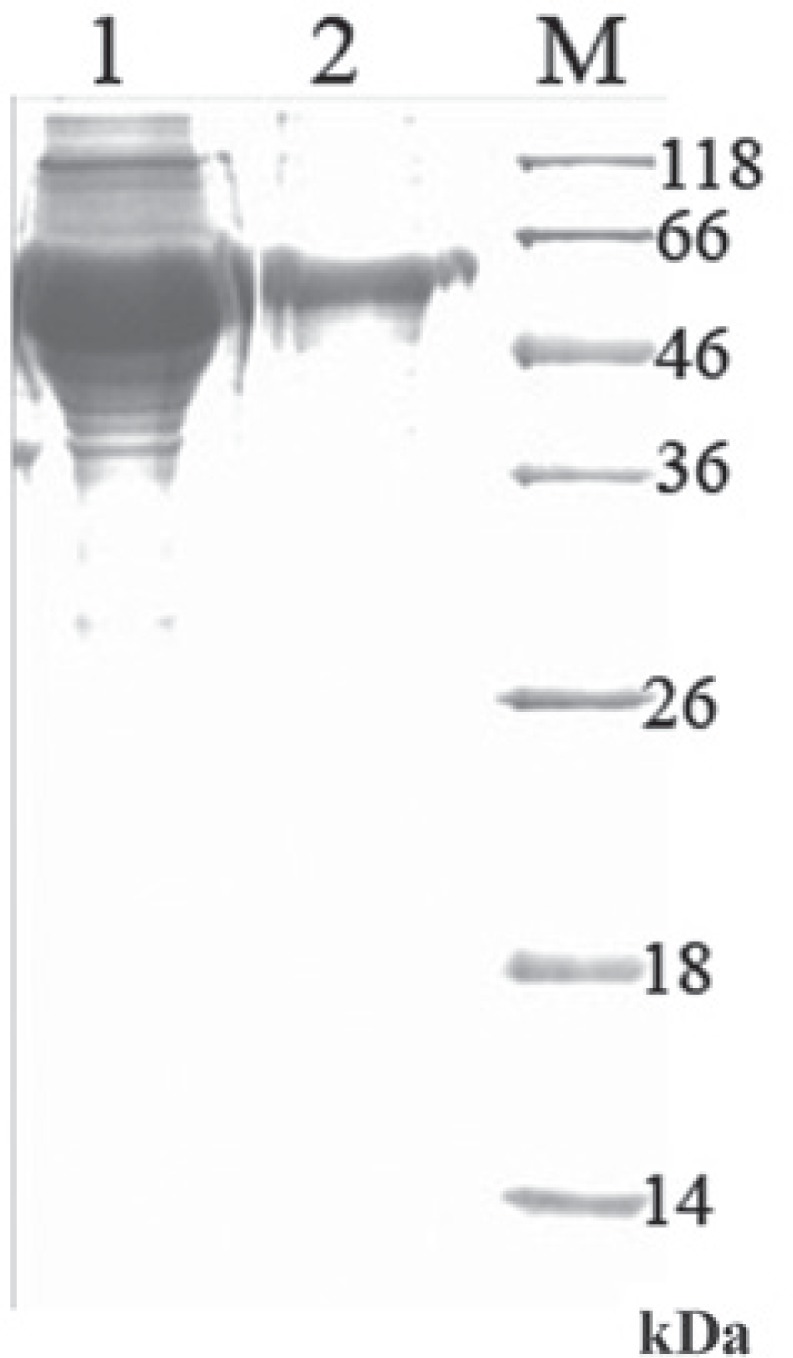
Purification of the diabody by molecular sieve chromatography (10% SDS-PAGE). Lane 1, target protein prior to purification by molecular sieve chromatography; lane 2, target protein purified by molecular sieve chromatography; lane M, protein marker. SDS-PAGE, sodium dodecyl sulfate-polyacrylamide gel electrophoresis.

The diabody (∼56 kDa) was successfully expressed in a small amount with the induction of IPTG (Fig. 5, lane 2), and was identified in the supernatant (Fig. 5, lane 3) and precipitate (Fig. 5, lane 4) of bacterial lysates. As the diabody mainly existed in the supernatant of bacterial lysates, it was a soluble protein. Following affinity chromatography using the Ni-NTA column, one main band with the molecular weight of 56 kDa existed in the 30 and 500 mmol/l imidazole eluates, yet the majority of the diabody was located in the 30 mmol/l imidizole eluate (Fig. 5, lanes 6 and 7). However, with miscellaneous protein contamination, the recombinant protein was not pure enough; therefore, it required further purification. Following purification by molecular sieve chromatography, the target protein presented one single band in SDS-PAGE (Fig. 6). As a result, we obtained the electrophoretically pure recombinant protein. The purity was >90% and the concentration was 1.65 mg/ml.

### Binding specificity of the recombinant protein

**Figure 7.**
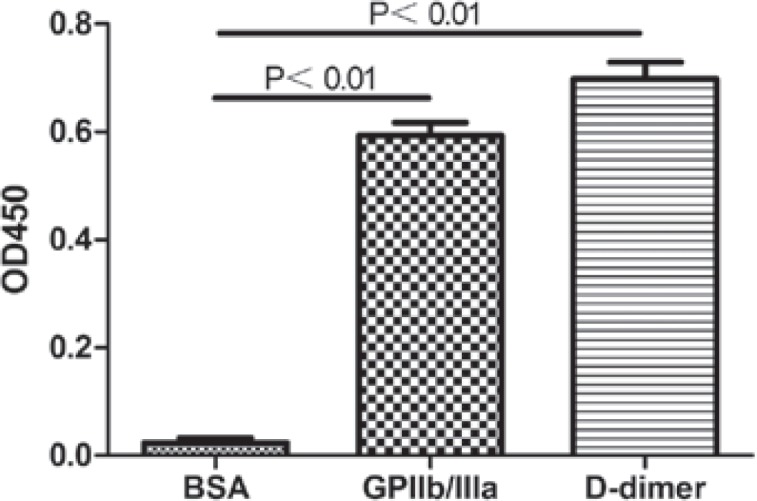
Binding specificity of the recombinant protein. Data are presented as mean ± SD. The statistical comparisons were made by t-test and differences were considered to be significant at P<0.05. BSA, bovine serum albumin.

The purified recombinant protein was shown to be specific for GPIIb/IIIa and D-dimer since there was no cross-binding to other proteins, including BSA (Fig. 7).

## Discussion

Fibrin is a clear target for antithrombotic or fibrinolytic agents. Sufficient amounts of fibrin are present even in platelet-rich thrombi. Certain types of fibrin-targeted anticoagulants have been produced. Antibodies, including MA-15C5, directed against the D-dimer fragment of cross-linked human fibrin, have been fused to recombinant scu-PA and used successfully to target clots (10). A study in various *in vivo* models of venous thrombosis has demonstrated that thrombolysis by 59D8-scuPA is significantly faster and more potent compared with that by the clinically used urokinase (11). Another fibrin-targeted anticoagulant was successfully developed by fusing hirudin to the generated fibrin-specific scFv of 59D8 to target a developing clot (12). Moreover, studies concerning platelet-targeted anticoagulants have also been reported (13,14). In one study, an anti-GPIIb/IIIa single-chain antibody was genetically fused with a potent, direct factor Xa (fXa) inhibitor and tick anticoagulant peptide (TAP) (15). However, these chimeric proteins target only one portion of the thrombus: fibrin or platelets. Thrombolytics that targeted fibrin and platelets simultaneously may have enhanced potency and clot specificity. A bispecific antifibrin-antiplatelet urokinase conjugate (BAAUC) was created by coupling urokinase to the monovalent Fab’ from the antifibrin monoclonal antibody 59D8 and the monovalent Fab’ from the anti-glycoprotein GPIIb/IIIa monoclonal antibody 7E3 (16). *In vitro*, this bispecific drug has the potency to lyse fibrin-rich and platelet-rich thrombi with high efficacy and to effectively inhibit platelet aggregation. However, for penetrating into the thrombus, a target-oriented thrombolytic agent with a smaller molecular weight than BAAUC is required. Moreover, the majority of monoclonal antibodies or Fab’ portions in recombinant proteins are derived from mice and may produce human anti-mouse antibodies (17). Thus, we aimed to construct a new and effective anticoagulant or thrombolytic agent with a small molecular weight, low immunogenicity, strong tissue penetrating force and a good specific binding capacity for thrombi.

As the scFv is the smallest antibody fragment with a complete antigen-binding site (18), we used scFv molecules specific for D-dimer and GPIIb-IIIa to construct the diabody. This construct is smaller than those containing whole Fab’ fragments and has improved thrombus penetration. Furthermore, the anti-D-dimer and anti-GPIIb-IIIa scFvs are from a fully human single-chain Fv library; therefore, the recombinant diabody is a human antibody.

In our study, we used a non-standard method to construct bispecific antibodies. Since Holliger *et al* (19) invented the diabody by cross-linking the genes of the heavy-chain and light-chains of the variable regions of two antibodies to form a hybrid scFv, the majority of diabodies have been created by restriction enzyme digestion and ligation (20,21). In a change from the common method, we used blunt-end ligation to generate the recombinant plasmid. As the gene sequences of the anti-D-dimer and anti-GPIIb-IIIa scFvs are known, using the circular plasmids as templates, the gene of anti-GPIIb-IIIa scFv was conveniently amplified and inserted into the vector pIT2, which already contained the anti-D-dimer scFv (Fig. 2). PCR and gene sequencing demonstrated that a new plasmid of the diabody in the A1VH-A1VL-G9VH-G9VL orientation was successfully constructed. In this method, high fidelity PCR is the crucial step, particularly since the amplified product (construct I) was particularly long at ∼4,950 bp (Fig. 3). However, the superiority of this blunt-end ligation method is the reduction in the number of processes for generating the recombinant plasmid due to the lack of restriction enzyme digestion steps, which is convenient for researchers.

In our recombinant plasmid, the two scFv genes share only lac promoter and terminator without mutual influence. After eliminating the repression function of lac by IPTG, a double-specific (scFv)_2_ fragment was expressed in HB2151 cells. Linking the two scFvs tandemly with a linker is possibly the simplest way to keep two scFv together as bispecific molecules, and is likely to avoid undesired associations. As the molecular weight of the A1-scFv or G9-scFv monoclonal antibody is 28 kDa (6,7), the expected molecular weight of the double-specific (scFv)_2_ fragment is 56 kDa, which was verified by SDS-PAGE analysis (Fig. 5). Diabodies represent a class of bispecific antibody fragments similar in size to a Fab fragment (22). Their small size potentially gives them access to tissues such as thrombi that are poorly accessible to intact antibodies, allows rapid clearance from blood and non-targeted tissues and lowers the immunogenic response.

The ELISA results demonstrated that the new D-dimer and GPIIb/IIIa single-chain bispecific antibody was able to simultaneously identify and bind two specific antigens (Fig. 7). Whether it is possible to apply this diabody *in vivo* requires further experimental investigation. If it presents a good specific binding capacity for platelets and fibrin *in vivo*, the diabody itself may be applied as an antiplatelet agent or be conjugated with a thrombolytic drug for research into its target-oriented thrombolytic function *in vitro.* Therefore, an ideal target-oriented thrombolytic drug may be produced. With the advantages of a lower molecular weight, higher antigen-binding power and comparatively lower immunogenicity, it is likely to have a high specificity and functional avidity for thrombi.

## Figures and Tables

**Table I.** Primers for polymerase chain reaction.

Primer name	Sequence
vla^−^	CCG TTT GAT TTC CAC CTT GGT
Vector^+^	GCG GCC GCA CAT CAT CAT CAC CAT CA
linker^+^vlb^+^	GGG GGC GGG GGA TCA ATG GCC GAG GTG CAG CTG T
vlb^−^	CCG TTT GAT TTC CAC CTT GGT CCC TTG

The underlined sequence is the site of the glycine-serine linker.

## References

[1] Cho J, Mosher DF (2006). Enhancement of thrombogenesis by plasma fibronectin cross-linked to fibrin and assembled in platelet thrombi. Blood.

[2] Bennett JS (2005). Structure and function of the platelet integrin alpha-Ibbeta3. J Clin Invest.

[3] Davì G, Patrono C (2007). Platelet activation and atherothrombosis. N Engl J Med.

[4] Bode C, Runge MS, Schönermark S (1990). Conjugation to antifibrin Fab’ enhances fibrinolytic potency of single-chain urokinase plasminogen activator. Circulation.

[5] Peterson E, Owens SM, Henry RL (2006). Monoclonal antibody form and function: manufacturing the right antibodies for treating drug abuse. AAPS J.

[6] Xia HL, Tan Z, Chen DJ, Qiao JG, Qiu RF (2011). Isolation of specific humanized anti-D-dimer scFv fragments from scFv phage libraries. Zhonghua Wei Sheng Wu Xue He Mian Yi Xue Za Zhi.

[7] Xia HL, Tan Z, Chen DJ, Qiao JG, Qiu RF (2011). Production of monoclonal anti-GP II b/IIIa scFv antibodies from scFv phage libraries. Chin J Exp Surg.

[8] Dincq S, Bosman F, Buyse MA (2001). Expression and purification of monospecific and bispecific recombinant antibody fragments derived from antibodies that block the CD80/CD86-CD28 costimulatory pathway. Protein Expr Purif.

[9] Jongmans W, van den Oudenalder K, Tiemessen DM (2003). Targeting of adenovirus to human renal cell carcinoma cells. Urology.

[10] Holvoet P, Laroche Y, Stassen JM (1993). Pharmacokinetic and thrombolytic properties of chimeric plasminogen activators consisting of a single-chain Fv fragment of a fibrin-specific antibody fused to single-chain urokinase. Blood.

[11] Dewerchin M, Vandamme AM, Holvoet P (1992). Thrombolytic and pharmacokinetic properties of a recombinant chimeric plasminogen activator consisting of a fibrin fragment D-dimer specific humanized monoclonal antibody and a truncated single-chain urokinase. Thromb Haemost.

[12] Peter K, Graeber J, Kipriyanov S (2000). Construction and functional evaluation of a single-chain antibody fusion protein with fibrin targeting and thrombin inhibition after activation by factor Xa. Circulation.

[13] Okabayashi K, Tsujikawa M, Morita M (1996). Secretory production of recombinant urokinase-type plasminogen activator-annexin V chimeras in *Pichia pastoris*. Gene.

[14] Jiang P, Changgeng R, Ru B (2000). Construction and expression of antibody targeted plasminogen activator*. Enzyme Microb Technol.

[15] Stoll P, Bassler N, Hagemeyer CE (2007). Targeting ligand-induced binding sites on GPIIb/IIIa via single-chain antibody allows effective anticoagulation without bleeding time prolongation. Arterioscler Thromb Vasc Biol.

[16] Ruef J, Nordt TK, Peter K, Runge MS, Kübler W, Bode C (1999). A bispecific antifibrin-antiplatelet urokinase conjugate (BAAUC) induces enhanced clot lysis and inhibits platelet aggregation. Thromb Haemost.

[17] Tcheng JE, Kereiakes DJ, Lincoff AM (2001). Abciximab read-ministration: results of the ReoPro Readministration Registry. Circulation.

[18] Leath CA, Douglas JT, Curiel DT, Alvarez RD (2004). Single-chain antibodies: A therapeutic modality for cancer gene therapy (review). Int J Oncol.

[19] Holliger P, Prospero T, Winter G (1993). “Diabodies”: small bivalent and bispecific antibody fragments. Proc Natl Acad Sci USA.

[20] Liu F, Chen Z, Jiang W, Yang C, Xiong D, Zhu Z (2008). Improvement in soluble expression levels of a diabody by exchanging expression vectors. Protein Expr Purif.

[21] Stamova S, Cartellieri M, Feldmann A (2011). Unexpected recombinations in single chain bispecific anti-CD3-anti-CD33 antibodies can be avoided by a novel linker module. Mol Immunol.

[22] Kriangkum J, Xu B, Nagata LP, Fulton RE, Suresh MR (2001). Bispecific and bifunctional single chain recombinant antibodies. Biomol Eng.

